# IMPEC: An Integrated System for Monitoring and Processing Electricity Consumption in Buildings

**DOI:** 10.3390/s20041048

**Published:** 2020-02-14

**Authors:** Mohamed Aymane Ahajjam, Daniel Bonilla Licea, Mounir Ghogho, Abdellatif Kobbane

**Affiliations:** 1 TICLab, International University of Rabat, Rabat 11100, Morocco; aymane.ahajjam@uir.ac.ma (M.A.A.); daniel.bonilla-licea@uir.ac.ma (D.B.L.); 2 ENSIAS, Mohammed V University, Rabat BP 713, Morocco; abdellatif.kobbane@um5.ac.ma; 3 School of EEE, University of Leeds, Leeds LS2 9JT, UK

**Keywords:** energy monitoring, Non-Intrusive Load Monitoring, disaggregation

## Abstract

Non-intrusive Load Monitoring (NILM) systems aim at identifying and monitoring the power consumption of individual appliances using the aggregate electricity consumption. Many issues hinder their development. For example, due to the complexity of data acquisition and labeling, datasets are scarce; labeled datasets are essential for developing disaggregation and load prediction algorithms. In this paper, we introduce a new NILM system, called Integrated Monitoring and Processing Electricity Consumption (IMPEC). The main characteristics of the proposed system are flexibility, compactness, modularity, and advanced on-board processing capabilities. Both hardware and software parts of the system are described, along with several validation tests performed at residential and industrial settings.

## 1. Introduction

The smart grid concept consists of shifting from a centralized energy generation scheme to a fully-automated and distributed power generation network that provides a two-way flow of electricity and information between utilities and end-users [1]. In this context, it has been shown that providing regular feedback to users about their electricity consumption may help them reduce the electricity bill [2]. Such a regular feedback also provides utility companies with the data needed to understand the consumers’ behaviors and thus bring the electricity demand and supply closer to a perceived optimum [3]. This could be achieved using data acquisition and monitoring techniques. In fact, data acquisition is widely applied to collect data describing phenomena in various fields from healthcare and wellbeing [4] to solar energy [5]. Additionally, data monitoring is becoming more and more essential in order to make and provide data-driven decisions and services in ever-growing fields such as smart buildings [6].

However, monitoring the energy consumption of each electrical appliance requires generally intrusive techniques. An alternative approach is to extract this information from the aggregated data; this is referred to as non-intrusive load monitoring (NILM). NILM is a process that was first developed in the 1980s by F. Schweppe and G.W. Hart [7]. A system implementing the NILM technique is based on a single sensor connected to the entry point of a household. Accordingly, this system measures the aggregated power signals which are later analyzed to identify the activity of the individual household appliances.

In some papers, for example, Reference [8], NILM and disaggregation are terms used interchangeably. But as Abubakar et al. point out in Reference [9], these terms should be differentiated; NILM is a process that can be used to achieve the goal of disaggregating the overall electricity usage at utility service entry [9]. It was developed as an alternative to intrusive load monitoring which requires a power meter connected to each and every appliance and to a central metering point to perform the same task of power disaggregation and load monitoring [10]. The use of such an approach allows the energy provider and the consumer to gain information inexpensively to make informed decisions and serve as a base for other tasks such as load forecasting [11].

A NILM process can be roughly divided into three main sub-processes:

- Electric signals acquisition: this is the core task of NILM. It consists of measuring the aggregated electricity consumption of loads (i.e., voltage and current signals) from the utility service entry (also called whole-premises consumption).
- Feature extraction: this task consists of retrieving useful information from the acquired waveforms, such as active and reactive powers, RMS values or harmonics of signals, among others [12].
- Load disaggregation: using the features extracted, a load disaggregation process is performed by applying algorithms to aggregated consumption datasets of load signatures [13].

Electrical energy monitoring has recently known a significant development, for instance, many systems have been developed for acquiring whole-household power consumption either based on direct sensing, indirect sensing or both. Direct sensing refers to the measurement of electrical signals directly from the source using power measuring sensors (i.e., current and voltage sensors). On the other hand, indirect sensing refers to the indirect measurement of electrical power consumption by measuring non-electrical signals related to the consumption; see Table 1 for more information.

In the context of indirect sensing, some researchers have used a combination of radio-enabled sensors (acoustic, magnetic, etc.) communicating with a central hub to estimate the consumed power. For example, in Reference [14], Kim et al. constructed a power monitoring system called ViridiScope, based on indirect sensing of both sound and magnetic field variations coming from individual appliances via inexpensive sound sensors, estimating the end-point power consumption within a 10% error. In Reference [15], the authors used improved inexpensive contactless electromagnetic field (EMF) sensors for loads’ state detection. All of these systems rely on using one sensor and one transmitter for every appliance (i.e., intrusive load monitoring). In Reference [18], Amac et al. built an energy monitoring system based on a wireless sensor network, that reports device-level (residential appliances) power consumption using acoustic signatures detected via wireless sensors.

In the context of direct sensing, the authors of a more recent paper [20] have developed a NILM system (direct sensing) to collect high sampling frequency datasets of aggregated power measurement scenarios (up to six appliances working simultaneously). Another system, specific to control appliances’ turn on and off time instants, was developed and coupled with the NILM system to analyze the corresponding transients of appliances. In addition to the computer (running LabVIEW) storing data and sending operating signals to an acquisition card coupled with current and voltage sensors, a system based on a processor was used in order to execute the pre-loaded measurement scenarios (stored in SD card) controlling the state of connected appliances.

Systems based on both sensing approaches have also been developed and used to construct datasets of whole-house or singular appliances’ power consumption at high and low sampling rates. In Reference [16], a system was installed in several houses enabling the collection of whole-house data sampled at 15 kHz. It comprises oscilloscopes connected to outlets, current sensors coupled to power mains and an off-the-self energy monitoring system (eMonitor: off-the-self energy monitoring and management system developed by Powerhouse Dynamics) and a laptop where all data is received, logged in an external hard drive and sent to a central server. In Reference [17], Kyle et al. succeeded to build a NILM based system that enabled the collection of a whole-house dataset with labeled activity sampled at 12 kHz over a whole week. A National Instrument (NI) USB (NI USB-9215A data acquisition device) handled the collection of aggregated data from voltage and current signals coming from a step-down voltage transformer and two split-core current transformers at 12 kHz, while a computer handled the operation of logging. In addition, in parallel to collecting appliance state transitions, wireless sensor networks were designed for collecting environmental data (light level, sound intensity, humidity, vibration, etc.) and plug-level power consumption data (sampled at 1 kHz). Yet, for appliances that are difficult to monitor using the prior system, a NI USB coupled with current transformers was used to acquire mains’ sub-circuits consumption. Details on the aforementioned systems are shown in Table 1.

On a related note, recent research studies have focused on challenges relating big data generation and management in multi-functional environments such as smart buildings. For instance, the researchers in Reference [21] propose the use of a tag-based wearable solution to gather occupant-specific localization information for an automated labelling of activity sensor data to occupants in multi-residential smart homes. In fact, the proposed solution takes advantage of Bluetooth Low Energy (BLE) technology in order to broadcast the occupant’s unique identification at specific time intervals and with a low energy footprint. In Reference [22], the authors proposed a global and comprehensive layered architecture for future smart homes that deals with all sensed data coming from data-driven internet-of-things (IoT) systems of different smart home applications (i.e., healthcare, energy consumption, etc.). Several other features were added to the proposed architecture handling security (i.e., regulations of user access and data exchange processes) and IoT device interoperability (i.e., a common standard for data format and exchange). Similarly, the authors of Reference [6] have proposed and implemented another layered architecture for smart homes, but incorporating practical healthcare and wellbeing services (i.e., monitor occupants vital signs, remote consultation with a physician), energy management services (e.g., hybrid AC/DC platform for sustainable energy generation, controllable IoT devices), in addition to security services (e.g., user authentication).

**Figure 1:**
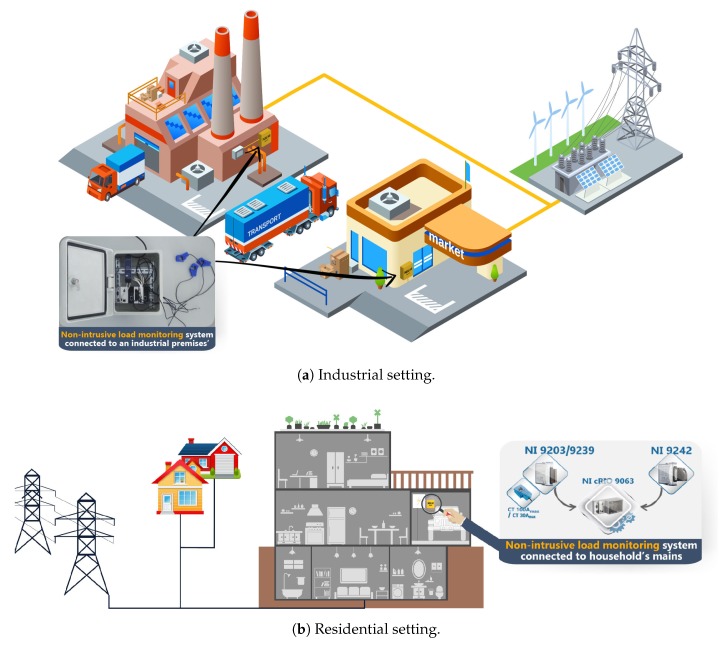
Illustrations of the Integrated Monitoring and Processing Electricity Consumption (IMPEC) systems installed in different premises.

In this paper, we present an integrated NILM system that can acquire, process and log both whole-premises power consumption and load signatures. The proposed system, coined IMPEC, is suitable for deployment in residential or industrial setting (see Figure 1). The flexibility of the proposed system, ensures the efficient execution of the first two NILM tasks (i.e., signal acquisition and feature extraction), allows the user to customize its components, and makes it easy to incorporate new tasks (e.g., disaggregation and forecasting). This system is based largely on NI equipment. It is able to collect and process voltage and current waveforms with a sampling frequency up to 50 kHz. It is non-intrusive because it connects to the premises’ power mains relying on direct analog voltage measurements and open (split) core Current Transformers (CT) for current measurements (CTs are a type of current sensors designed to produce an alternating current in its secondary winding proportional to the current being measured in its primary winding [23], while open core CTs are current transformers that have a distinctive opening which makes it easy to connect them to the load conductor or bus bar). With an easy to use graphical interface, the system can be easily controlled to operate as desired. Experimental results show the adequate run of our proposed system in collecting data and extracting features in real time.

The main contribution of this paper resides in the development of a new method to investigate electric signals, which is materialized through the functionalities of the proposed IMPEC system. The latter is endowed with the modularity, flexibility and advanced on-board processing capabilities required for research and development tasks in the field of smart grid, such as flexible data acquisition and data management, feature extraction, and ease of incorporating customized (by the user) functionalities, for example, load disaggregation, power quality disturbances detection, etc. As opposed to the aforementioned systems reported in the literature, our system does not require an external computer, hence making it suitable for field research. Further, the high flexibility of IMPEC allowing to control various system’s parameters, such as the sampling frequency, sets it apart from the other systems mentioned before.

The rest of the paper is organized as follows: Section 2 presents a hardware and software description of IMPEC. In Section 3, results from several tests in a residential and industrial settings are presented, followed by a discussion on IMPEC’s performance and encountered issues. In Section 4, an example of an incorporated task of load identification in IMPEC is presented. Section 5 concludes the paper.

## 2. System Description

We start this section by presenting some of the main properties of the proposed system:

- Compactness, modularity, simplicity to install (at premises’ mains), robustness and possibility to run in headless mode (operates without a display) or via a monitor. These characteristics make it suitable for acquisition tasks at residential and industrial settings.
- A user-friendly graphical interface offering meta-data logging describing the load, and premises.
- Effective collection, management, and processing of voltage and current waveforms at different high and low sampling rates (50 kHz maximum).
- On-board data processing and feature extraction (e.g., Active power, Reactive power, etc.) at user-defined rates and time slots.
- Data logging in local storage devices (e.g., USB stick or external hard drive), or can be transmitted via Ethernet cable or wirelessly.

Two versions of the IMPEC system were developed, one for single-phase electric loads and another for three-phase electric loads.

Now, we proceed to present a detailed description of its hardware and software components.

### 2.1. Hardware Description

**Figure 2:**
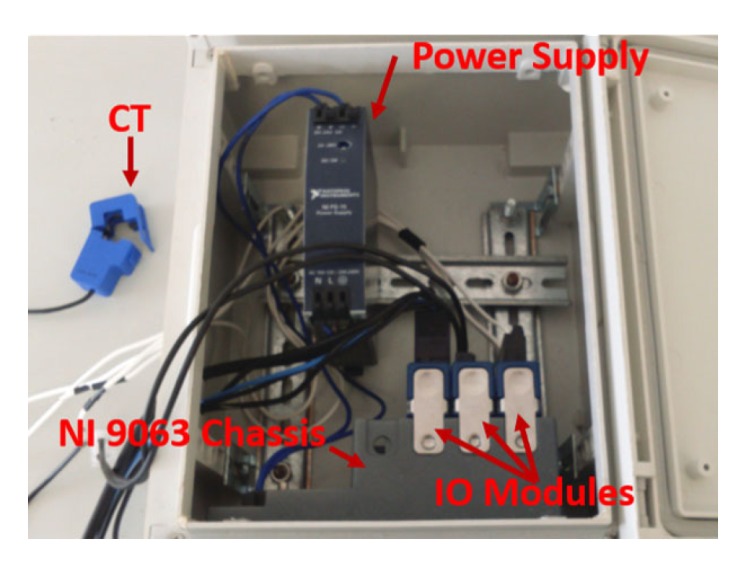
The IMPEC system with highlighted components in an enclosure box.

**Figure 3:**
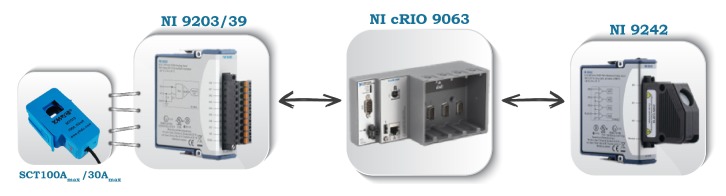
Basic components of IMPEC systems.

**Figure 4:**
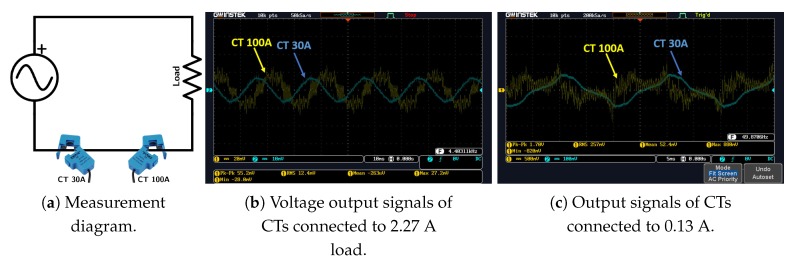
Comparison of Current Transformers (CTs) performance with two low current-demanding loads.

As it can be seen in Figure 2 and Figure 3, IMPEC has three principal components:

- NI compactRIO 9063 platform: it is the core component of the system. It houses a field-programmable gate array (FPGA) responsible for high speed measurements, a real-time controller and four IO slots (see Table 2) enough for multiple voltage and current acquisitions or wireless communication modules.
- NI IO Modules: to ensure minimum intrusiveness and maximum precision and accuracy, we opted to use NI 9242 [24] for direct voltage measurements which is a suitable choice given that it has a neutral line input alongside three analog inputs making it possible to have single-phase or three-phase measurements. For current measurements, we use NI 9239 [25] appropriate for voltage output CTs, or NI 9203 [26] appropriate for current output CTs (see Table 2) at sampling rates ranging from 1 kHz up to 50 kHz.
  The reason behind adopting two distinct IO modules for current measurements is to guarantee an independent use of the system regardless of the chosen current sensor’s type.
- Current sensor: a number of split core (or open core) current transformer with current or voltage output can be used for current measurements (i.e., 3 CTs are used for a three-phase IMPEC, 1 CT for the single-phase). In fact, a split core CT can be easily hooked to an existing wiring without disrupting the consumption at the premises’ mains, contrary to a solid core current transformer, which has a fixed opening and a lower accuracy when compared to the former type [27]. For the time being, both IMPEC systems use SCT013 sensors from YHDC [28]. They are a series of split core (non-invasive) current transformers that have a 13 cm opening diameter (appropriate for cables found in Moroccan buildings’ mains) and that come in different input ranges. Based on preliminary measurements in residential settings, the use of a current sensor with a rated input of 100 A seems more appropriate for large households with many electric loads, where the current can reach 70 A. Similarly, a CT of 30 A rated input is more appropriate for apartments with lower power consumption. On one hand, The SCT013-000 sensor has a high rated input but performs poorly when measuring current amplitudes less than $7A_{rms}$. On the other hand, sensor SCT013-030 has a lower rated input, but a higher sensitivity because it can measure (without significant distortion) amplitudes down to $2A_{rms}$. Indeed, as can be noticed in the measurements in Figure 4b,c, obtained as shown in the diagram of Figure 4a, the SCT013-030 sensor succeeds at giving a better representation of low amplitude current signals (i.e., lowest total harmonics distortion) compared to SCT013-000.

### 2.2. Software Description

A clear vision is essential to the success of a software project [29]. As stated in several publications, the essence of software engineering revolves around the same principles of problem solving: understanding the problem and need, planning a solution, carrying out the plan, and examining the results for accuracy [29].

**Figure 5:**
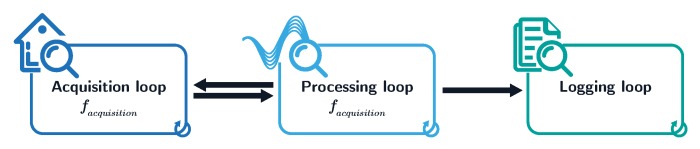
Main loops governing the IMPEC standalone application.

Toward providing a rich and valid data describing the electricity consumption in Moroccan buildings, the IMPEC system was developed to achieve three main functionalities, which are acquisition, processing and logging of electricity signals and related features, while being resistant to errors and flexible to the user’s needs. Consequently, a standalone application was developed using LabVIEW. It performs several automated tasks, which are mainly: voltage and current signal acquisition, lossless data transfer, accurate feature extraction, and secure and optimized data logging. All of these tasks are structured into three main loops. While both acquisition and processing loops run at a user-defined rates, with the acquisition’s rate ($f_{acquisition}$) always higher than the processing’s rate ($f_{extraction}$), the logging loop runs independently whenever data are available, minimizing data loss and optimizing error handling (see Figure 5).

**Figure 6:**
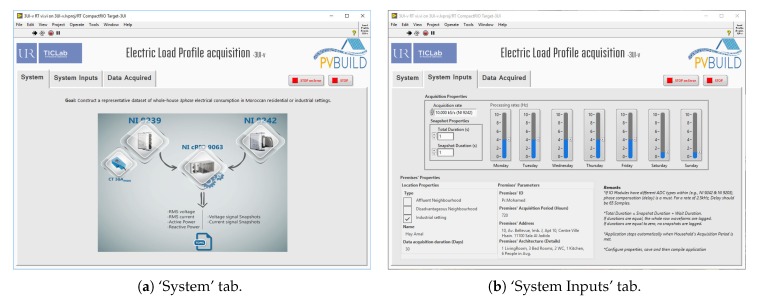
User interface of the three-phase electric load profile acquisition application.

**Figure 7:**
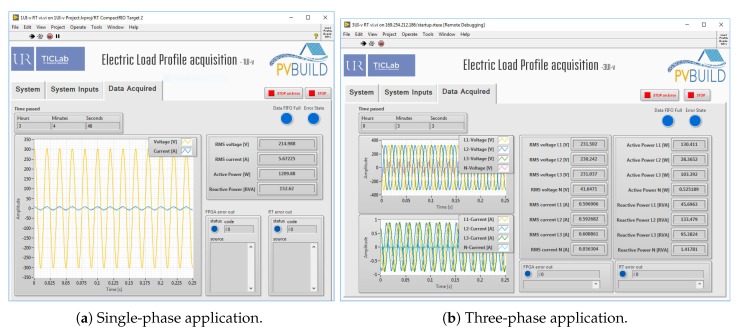
User Interfaces of the electric load profile acquisition applications running under several parameters.

The graphical user interface (GUI) accompanying the developed application (see Figure 6 and Figure 7) contains three tabs:

- ‘System’: it presents the hardware components of the IMPEC system (Figure 6a).
- ‘System Inputs’: as shown in Figure 6b, this is where the user specifies properties of the required acquisition task, and of the premises where the task is to be performed. In addition, this tab provides other controls such as a numerical variable to specify the duration of raw waveform snapshots to log, and seven vertical slide controls to define data processing rates at different time slots (i.e., every day of the week), providing control over sensitivity and bandwidth.
- ‘Data Acquired’: Captured data can be visualized in real-time in this tab (i.e., voltage and current waveforms, extracted features, elapsed time, and error LEDs) (see Figure 7).

**Figure 8:**
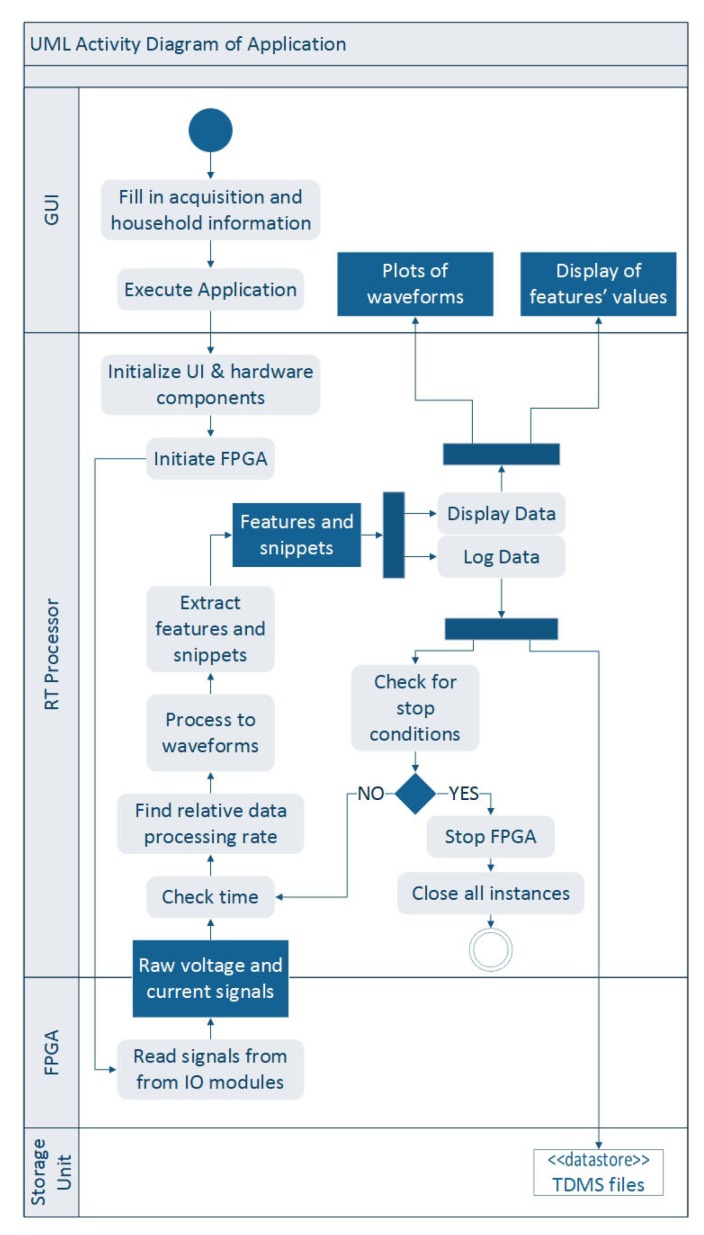
UML activity diagram.

The application’s Unified Modeling Language (UML) activity diagram is shown in Figure 8. It partitions the actions performed by the IMPEC system according to its components: GUI, Real Time Processor, FPGA and Storage Unit.

Given that the system is connected to the premises’ mains, the user has to provide the acquisition, premises and location properties through the ‘System Inputs’ tab of the application. This information is very important in the collection of a meaningful dataset for future analysis. The IMPEC system can then start operating. First, the Real Time Processor initializes all GUI and hardware components that are connected to the system. Second, it signals to the FPGA to start running its already compiled code, collecting raw signals coming from IO modules and transferring them to the processor via a FIFO memory. Using the user-defined rate of feature extraction, raw signals are then processed in order to get the appropriate current and voltage waveforms. Finally, specific features are then extracted and logged in specific TDMS files, alongside snapshots of waveforms in a storage unit.

Additionally, the Real Time Processor checks for stopping conditions (i.e., error in FPGA code, error in Real Time Processor code, acquisition user-defined duration reached) at the end of every cycle (i.e., read FPGA data and process them to extract features and snapshots) in order to maximize reliability of the application. The process of data logging is performed separately since operations on TDMS files may take a longer time to execute compared to the time required to read and process a chunk of data.

### 2.3. Signal Acquisition

As mentioned before, the FPGA is the component responsible for the collection of signals coming from IO modules through the platform’s chassis. Due to the fact that the sampling rate affects directly the energy consumed and the data quality [30], a compromise is needed, especially when storage capacity is limited.

#### 2.3.1. Energy Consumption

**Figure 9:**
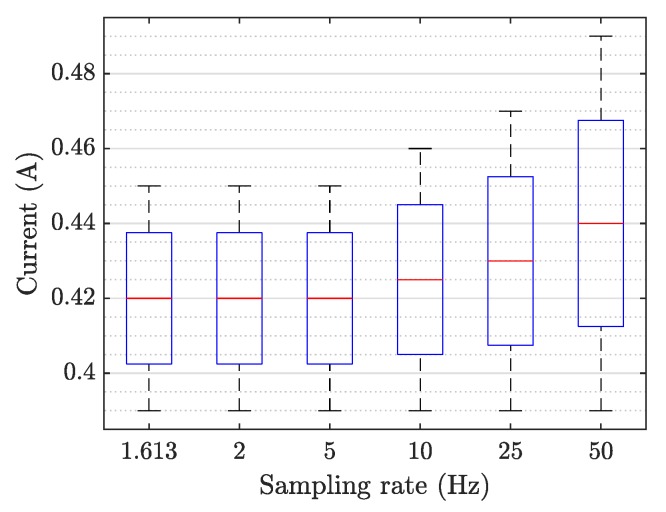
Whisker and box plot of the current consumed by the single-phase IMPEC system connected to a storage unit and operating at different sampling rates.

To investigate the energy consumption of the IMPEC system, we recorded the current consumed by the system under various sampling rates and a constant feature extraction rate (4 Hz) (see Figure 9).

In the operating state (i.e., system is turned on, sampling and storing data), when the sampling rate is <10kHz, the current consumed fluctuates around a mean value of 0.42A (∼92.4W). At higher sampling rates (≥10kHz), the current increases and reaches values of 0.49A (∼107.8W). In the idle state (i.e., system turned on with no ongoing task), the energy consumption is constant and is equal to 0.33A (∼72.6W) when no storage unit is connected and 0.39A (∼85.8W) otherwise.

To analyze the reported results, we refer to the two usage scenarios possible of IMPEC. In one hand, for whole-house acquisition (i.e., when the system is connected to the mains of a premises), the system would be always supplied with enough power to execute the task at hand. In another hand, for load signature acquisition

In general, the energy consumption of the system increases with the sampling rate. Since the system might be connected to the mains of a household and also for the sake of non-intrusiveness, it is of interest to reduce as much as possible the energy consumption of the system while maintaining a good performance. In any event, the user can control energy consumption by adjusting the sampling rate in the system’s interface.

#### 2.3.2. Data Synchronization

A phase shift between voltage and current signals represents an important monitoring feature in power quality analysis [31] and is essential for the extraction of several descriptive features such as the active and reactive powers. Hence, it is important to maintain the synchronization of the voltage and current waveforms being sampled to ensure that the phase difference between both waveforms is accurately measured. Consequently, another factor to consider is data synchronization between measurement modules containing different Analog to Digital Converters (ADC).

**Figure 10:**
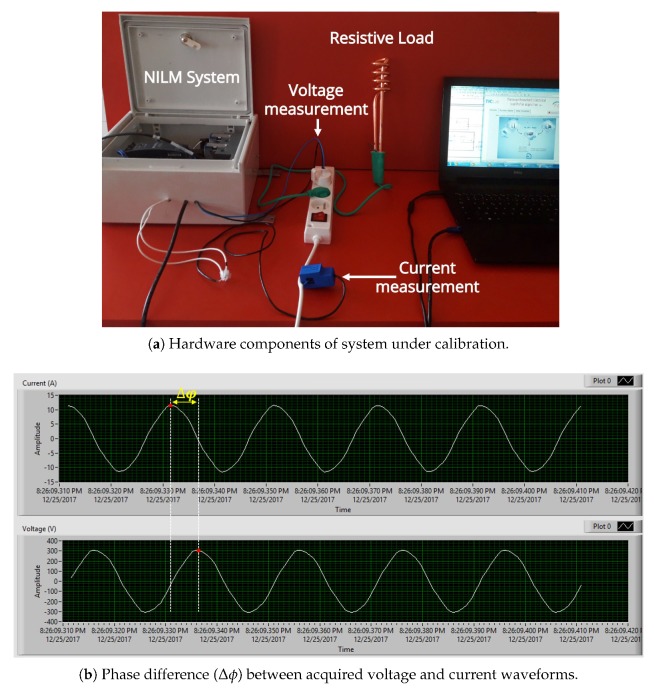
System calibration using a 2 kW resistive load.

When developing the system, the acquired voltage and current signals presented a temporal delay due to the hardware design. This delay was clearly identified during a calibration process, which consisted of testing the system with a pure resistive load of 2 kW (see Figure 10a).

Because of the nature of this load, the voltage and current waveforms are supposed to be in-phase. However, it is not the case as seen in Figure 10b. This delay needs to be compensated for by software in order to obtain accurate active and reactive power measurements. Note that this delay occurs when using IO modules with different types of ADCs, since their architectures/attributes differ from one another. Contrary to Successive-Approximation-Register (SAR) ADCs (housed in NI 9203 nodule), Delta-Sigma (ΔΣ) ADCs (housed in NI 9242 and NI 9239 modules) implement several additional components for oversampling, decimation filtering, and quantization noise shaping to achieve high resolution and excellent anti-aliasing filtering. The decimation filtering process, which averages and down samples data to produce an n-bit sample at a desired sampling rate, introduces a delay before the input signal is converted to a digital signal [32].

To mitigate this problem, a digital compensation method can be implemented. We obtained the best synchronization by introducing a digital FIR filter that delays the input signal by a specific number of samples when reading data in the FPGA side [33]. The number of samples needed to be delayed in order to synchronize IO modules can be computed using an appropriate formula (see Group delay in Table 2) that depends on the type of the IO module used and the acquisition’s sampling rate.

#### 2.3.3. Data Management

Data management is another crucial issue that we have tackled to make searching and analyzing data easier. Aside from the meta-data in TDMS files that include all information about the task, the system’s architecture and the premises properties, a specific design pattern (Producer/Consumer pattern) is used to ensure lossless data acquisition and transfer between the hardware components [34]. Moreover, upon meeting the acquisition duration condition that is specified by the user prior to starting the task, a stop function is signaled to all running functions so that all tasks and dependencies stop automatically.

It is worth noting that the system can be used to acquire appliance signatures (also called ground truths) and extracts rich features (e.g., transients [19], spectral traces [35]) that can be used for training disaggregation algorithms.

### 2.4. Feature Extraction

The transformation of large data into significant and representative features is essential, because it dictates the performance of the tasks to follow. Indeed, higher sampling frequencies give rise to a higher power consumption but provides more detailed signals and thus richer features enabling better performance (e.g., detection of a larger number of appliances [36]).

To extract more information from the voltage-current signals, a frequency analysis is desirable. Time-frequency analysis aims to find a representation that describes the temporal variations of the signals’ spectrum [37]. It is commonly performed by segmenting a signal into short periods, and estimating several parameters (e.g., its spectrum), using a sliding window [38]. Even though the application is flexible enough to define other features to be extracted from raw waveforms at lower rates, we chose to proceed with four temporal features, usually used in the literature [39], extracted at rates up to 10 Hz:

- RMS of voltage signal [V]
- RMS of current signal [A]
- Active Power [W]
- Reactive Power [VAR]

We have defined an interval of extraction rates between 1 Hz and 10 Hz by taking into account the fact that 10 Hz provide enough data to this type of applications and also by considering the existing trade-off between the quality of the captured signal and the storage space required to store it.

The system was designed to collect rich datasets either by adapting it to compute other temporal and spectral features online or offline (i.e., post-acquisition campaigns) using the four chosen features along with the raw signals (≤50 Hz) and their respective timestamps.

## 3. System Testing

To test the proposed IMPEC system, we have deployed it in Moroccan residential and industrial settings under various conditions (see Table 3). The single-phase system was deployed in a Moroccan household, see Section 3.1, while the three-phase system was deployed at a construction site at the International University of Rabat, see Section 3.2.

### 3.1. Single-Phase System Testing

The single-phase system was installed at the mains of a Moroccan middle-class $100\;m^{2}$ apartment of six people for several days.

**Figure 11:**
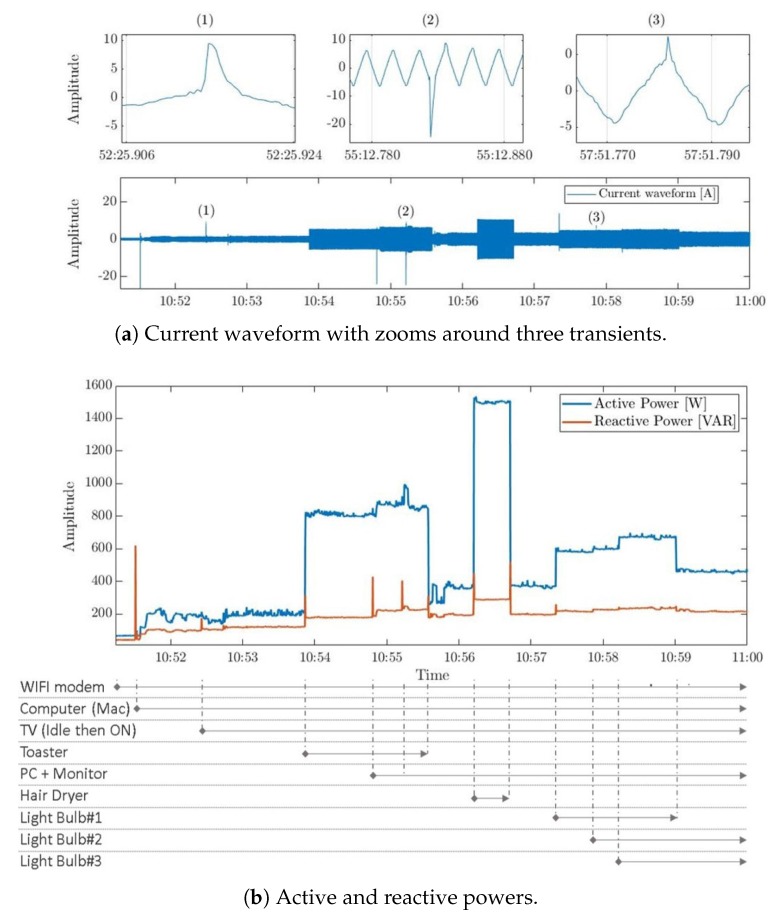
Whole-house power consumption with annotations.

First, the system was set to acquire the electricity consumption of several household appliances which were turned on and off over defined periods of time. The illustration of the acquired whole-house active and reactive powers in Figure 11b shows amplitude changes in both signals due to the state change of different appliances in use. On another hand, the system succeeded to capture transients of different lengths and amplitudes that appear in the reactive power as well as in the current waveform (see Figure 11a) whenever an appliance changes its state, due to its inductive component (e.g., Hair-dryer). Hence, this test confirms the ability of the designed system to capture the electricity consumption in detail, helping to collect different usage scenarios datasets that reflect real-life use of appliances.

**Figure 12:**
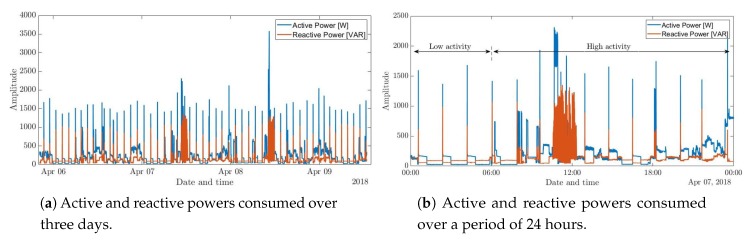
Snippets of electric power consumption acquired by the single-phase system in a residential setting over a period of seven days.

A second test was performed over a period of seven consecutive days at the same household without disturbing the daily lives of its residents. The electric consumption, seen in Figure 12, shows a periodic pattern; A higher activity and energy consumption can be observed around noon, while a lower and more stable consumption can be seen after midnight. This reflects the household residents’ daily routines, since family members are more active during the day than during the night. Note that in Figure 12b we observe some periodic spikes which are generated by the refrigerator which periodically switches between its cooling and idle states. This test shows that the developed system is able to execute monitoring tasks in a household setting for long periods of time without interruptions or nuisance to the premises’ residents.

The systems at hand can be used to acquire whole-premises electric consumption as well as to capture loads’ signatures. To test the latter function, the single-phase system was set to acquire signatures of eight commonly used appliances at the apartment. This time, the scenarios of loads usage (e.g., separate or combination of several loads running simultaneously) define the electric power circuit feeding loads to which the monitoring system would be connected. As for a whole-premises acquisition, each loads scenario description can be logged via the same fields found in the “system inputs” tab in the GUI of the related application. Chosen loads for monitoring are presented in Table 4.

**Figure 13:**
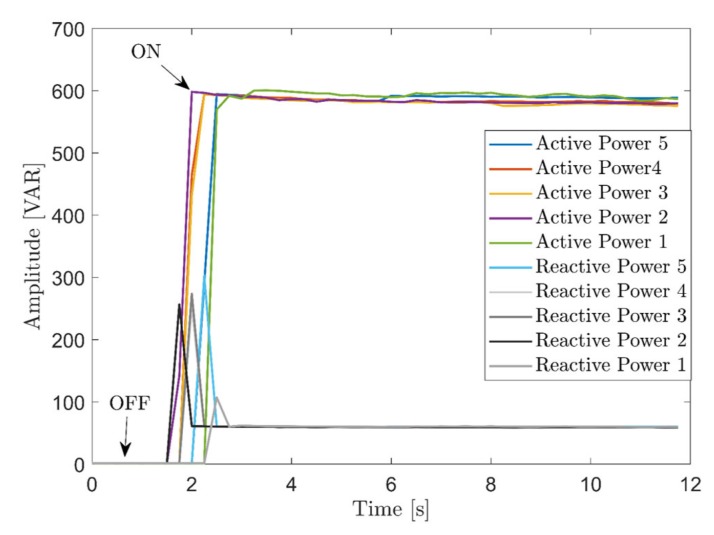
Example of acquired powers of an ON/OFF appliance.

The signature acquisition of separate loads scenarios (see Figure 13) was performed as follows: A two second pre-acquisition phase precedes every acquisition, to give the user enough time to turn on the load manually and ensuring the capture of the turn on transients of the appliances. Acquisition of every load’s state was limited to 10 seconds. With finite state machines (FSM), transition to a higher state were done after a one second wait at the current state. In total, every state was acquired five times since all appliances are controlled manually.

Appliances with an ON/OFF state are the easiest to monitor, since they have a single constant level of power consumed during their operation. An example of this type of appliances is the toaster as can be seen from its consumption pattern in Figure 13. As shown in the literature [40], transients are useful features for the task of loads’ identification and disaggregation.

**Figure 14:**
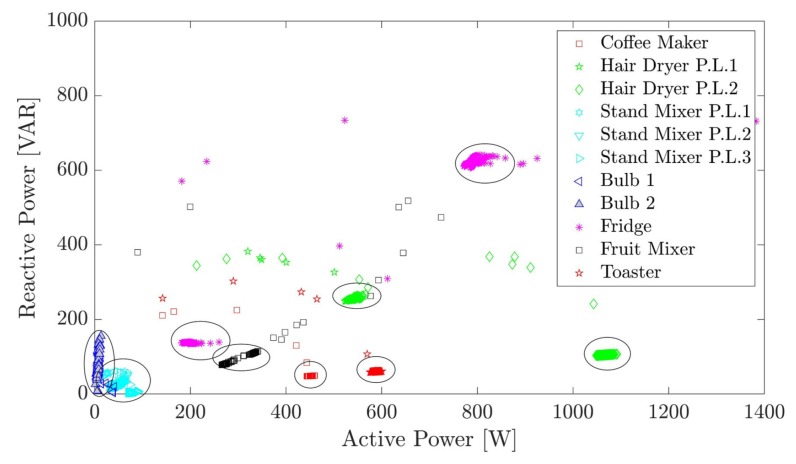
Appliances represented in the P-Q plane.

Another valuable feature for these tasks is the mapping of active and reactive powers in a 2D plane (called a P-Q plane) [41] (see Figure 14), where operational states of appliances can be clearly identified; the fridge, for instance, seems to have two distinct power states. Also, it can be seen clearly that the collected signatures of several appliances are visibly apart (e.g., Hair-dryer, fridge, coffee-maker, toaster, and fruit-mixer). However, the same cannot be said for appliances that consume little or similar instantaneous power (e.g., bulbs and stand-mixer) as they are harder to differentiate. Nevertheless, it has been proven in the literature [42], that the use of several features (i.e., steady-state P and Q, transients, etc.), in addition to the active-reactive 2D plane, does yield better disaggregation results.

### 3.2. Three-Phase System Testing

The three-phase system was installed for seven continuous days at a three-phase mains feeding a construction site, with several electric machines, located at the International University of Rabat, Morocco.

**Figure 15:**
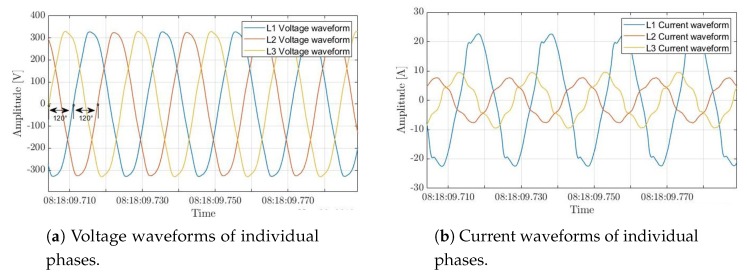
Snippets of voltage and current waveforms acquired by the three-phase IMPEC system in an industrial setting over a period of 7days.

**Figure 16:**
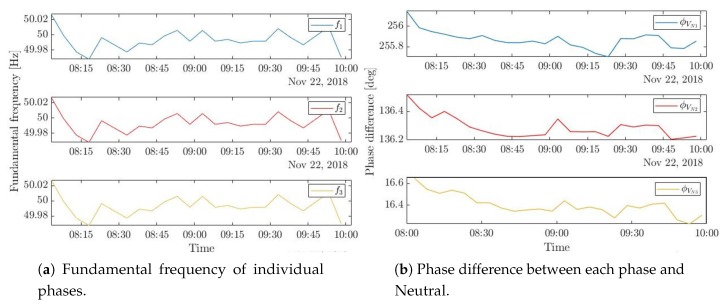
Fundamental frequency and phase difference of three-phase electricity consumption computed over a sliding window of five minutes.

In theory, the loads should be distributed evenly between the three phases. But in practice, it is uncommon in electric systems to have perfectly balanced loads, currents, voltages, etc. Indeed, based on the collected three-phase consumption of the construction site, it is clear that voltage waveforms of all phases are of equal magnitudes (see Figure 15a), and they oscillate at the same pace within a 0.04 Hz fluctuation of 50 Hz (see Figure 16a), which corresponds to the Moroccan mains frequency. In addition, there is a 120${}^{\circ }$ phase difference between each successive phases (see Figure 16b). Nonetheless, the current waveforms have different amplitudes flowing in the three phases, where most current is drawn from the first phase (see Figure 15b) indicating an unbalanced three-phase supply. This imbalance is probably due to using the three-phase supply to feed single phase loads, present at the construction site, with current drawn mainly from the first phase.

Based on this test, the three-phase system was validated to acquire and extract meaningful data describing the electricity consumption at a three-phase mains. Nevertheless, several problems were identified and solved during the testing phase, which mainly concerned the storage techniques with two distinct properties: the amount of data transferred from the FPGA memory side to the RT memory side and the amount of data held in a single file in the external storage unit. These issues were detected via specific errors prompted in GUI (e.g., FIFO full LED, Error indicators) halting the storage of captured data in TDMS files even if they are plotted in GUI figures. The first issue was addressed by specifying a RT side memory depth 10 times higher than the actual number of elements acquired (i.e., sampling frequency times the number of IO channels from where data is read). Since the file system of a storage device (e.g., NTFS, FAT32) dictates the maximum size of a single file that is possible to store, a size checking function of TDMS files was added to ensure the creation of new TDMS files for storage once the size of the previous ones has exceeded a certain threshold (e.g., 3GB).

## 4. Incorporation of a New Task in IMPEC: Load Identification

In this section we show how to incorporate new tasks into IMPEC. To achieve this, we implement within our proposed system an example of load classification algorithm. This is a task necessary to achieve non-intrusive load monitoring [43].

Load identification can be performed using either an event-based approach or an event-less approach. An event-based approach to solving a load identification problem relies on event-detection algorithms, which are implemented to search for state transition events (e.g., ON, OFF) occurring in the aggregated signal. Accordingly, load identification (i.e., appliance identification), can be achieved by classifying the found events according to their corresponding features. On another hand, an event-less approach does not rely on event-detection to perform load identification; it rather uses the extracted features directly as input for machine learning model learning. In the literature, there are different schemes to build such learning models (e.g., sequence-to-point, sequence-to-type) [43].

**Figure 17:**
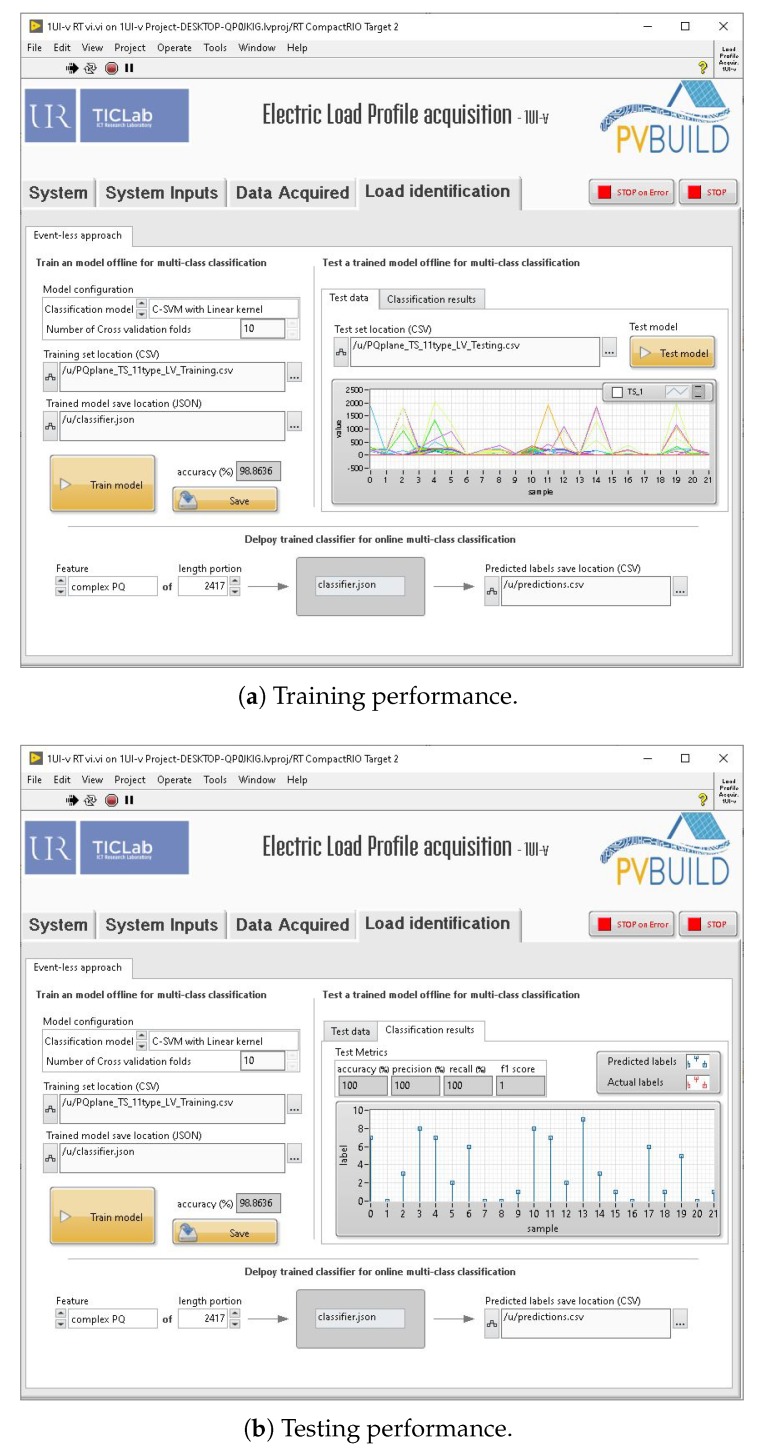
Training and testing of the Support Vector Machine (SVM) model for multi-class classification.

In this section, we choose to perform the load classification using an event-less approach, and more specifically a sequence-to-type scheme, which intends to train a model to identify the appliances producing the observed aggregated signal (i.e., the input of the model). Toward this end, another application was developed and then added to the IMPEC’s main application to facilitate two main tasks: model learning and deployment of the machine learning model for online load identification. The modified GUI is depicted in Figure 17. The GUI was developed to provide control to the user over the model’s training and testing steps:

- Classification model configuration: Support Vector Machine (SVM) or Artificial Neural Networks, in addition to their corresponding parameters (e.g., SVM’s kernel type).
- Learning datasets loading and processing: data presented as CSV files, where each row represents a signal and the last column represents its corresponding type (label).
- Model training and testing under the specified parameters and performance evaluation using four metrics (accuracy (1), precision (2), recall (3), *f*_1_ score (4)):
  (1) $$Accuracy=\frac{TP+TN}{TP+TN+FP+FN}$$
  (2) $$Precision=\frac{TP}{TP+FP}$$
  (3) $$Recall=\frac{TP}{TP+FN}$$
  (4) $$f_{1}=2\frac{Recall*Precision}{Recall+Precision},$$
  where, TP is the number of the true and positive cases, TN is the number of the true and negative cases, FP is the number of the false and positive cases, FN is the number of the false and negative cases in the data.
- Model saving (as a JSON file) for use in online load identification.

Once the model learning is performed, an online load identification can be attained by deploying the trained model for classification, similar to the model’s testing phase, but this time on a stream of continuous subsets of the acquired data with no labels. The start and end timestamps of each classified subset, as well as its predicted class, are logged in a CSV file once the task is finished.

We note that the incorporation of the online load identification capability in the IMPEC’s main application is done by placing its corresponding code in the system’s data logging loop where streams of data, with an equal length, are processed. The model’s learning capability is incorporated by placing the corresponding code as a separate structure once the Training button is activated.

To test the load identification capabilities added to the system, we trained a Support Vector Machine (SVM) model for multi-class classification using a cross-validation scheme and a training dataset containing a subset (80%) of the complex P-Q array of 11 home appliances (Blender, Hair curler, Hair straightener, Ironing machine, Oven, Vacuum cleaner, TV, Refrigerator, Washing machine, PS3, Water heater) previously acquired using the system (see Figure 17a).The testing of the model was performed using a testing dataset containing the rest (20%) of the acquired data and evaluated using the classification metrics (see Figure 17b).

We note that since the aggregated signals can contain the consumption of multiple loads at the same time, a multi-label model could provide better performance at load identification. The current version of IMPEC supports only multi-class classification; yet, one workaround to this constraint is to develop several load-specific binary models following a one-vs-all classification method. These models can be loaded and deployed simultaneously to infer the presence or absence of their specific loads in the aggregated signal. Now, we also have to mention that the training, testing of the model and its deployment can run on IMPEC or on a computer.

Finally, empowered by LabVIEW, other tasks, useful for research, can be integrated to the IMPEC system, such as event-based load disaggregation, time series forecasting, and/or power quality disturbance detection. Currently, as mentioned before, the system already has communication capabilities using Ethernet but wireless communication capabilities can also be added to transmit desired collected data to a server.

## 5. Conclusion and Future Work

In this paper, we introduced IMPEC, an integrated system for monitoring and processing electricity consumption of buildings. Taking advantage of NI hardware and software solutions, as well as other hardware components, this system is able to perform non-intrusively high-frequency acquisitions of voltage and current waveforms in residential and industrial settings. In addition, it can be used to collect appliance signatures and extract features in high and low sampling frequencies. One of its main features is its customizability to suit the user choices and particular needs, as well as its flexibility to add new capabilities such as load identification. Although the IMPEC system is characterized by a relatively high power consumption, it is endowed with many capabilities and provides high signal quality, making it a valuable tool for research. Finally, acquisition campaigns in residential and industrial settings using the single-phase and three-phase versions of the IMPEC system, respectively, are ongoing in an effort to collect fully labeled electricity consumption datasets, which could be used by the scientific community for different applications including load disaggregation. 

## Figures and Tables

**Table 1:** Some of the key electric energy monitoring systems in the literature.

Study	Year Published	Monitoring Type	Used Sensors	Used Features
[14]	2009	Indirect sensing	Sound sensors	Sound level, Light intensity,
			Light sensors	Std Dev of the magnetic field
			Magnetic sensors	
[15]	2010	Indirect sensing	Hertzian antenna	RMS of the magnetic field
			Magnetic field sensor	
[16]	2011	Direct sensing	Current and voltage sensors	AC waveforms
[17]	2012	Direct and Indirect sensing	Plug-level meters Voltage transformer Current transformer Circuit panel meters Environmental sensors	Humidity, Vibration, Light level, PIR motion, Sound intensity, Barometric pressure, Active and Apparent powers, RMS of voltage and current waveforms
[18]	2012	Indirect sensing	Acoustic sensors	Spectral flux, Spectral roll-off,
				Spectral-centroid, Band-level energy,
				Short-time energy, Zero-crossing rate,
				Mel-frequency cepstral coefficients
[19]	2016	Direct sensing	Current and voltage sensors	Voltage zero-crossing, Transients in voltage and current waveforms

**Table 2:** Parameters of IO Modules.

Parameter	NI 9242	NI 9239	NI 9203
Channels	3AI, 1N	4AI	8AI
Sampling rate	50 kHz/ch	50 kHz/ch	200 kHz
ADC resolution	24 bits	24 bits	16 bits

**Table 3:** Tests of single-phase and three-phase systems under different scenarios and acquisition parameters.

Test	System under Test	Setting	Target	Acquisition Period	Acquisition Rate	Extraction Rate
Test 1	Single-phase	Residential	Whole-house	10 mins	2.5 kHz	4 Hz
Test 2	Single-phase	Residential	Whole-house	7 days	12.5 kHz	4 Hz
Test 3	Single-phase	Residential	Individual load	10 s/power state/load	25 kHz	4 Hz
Test 4	Three-phase	Industrial	Whole-premises	7 days	5 kHz	6 Hz

**Table 4:** Properties of recorded loads.

Loads	Manufacturer	Rated Voltage	Rated Power	Power States
Stand mixer	Krups	220 V	140 W	FSM
Hair-dryer	Fagor	220 V	1000 W	FSM
Fridge	Sierra	230 V	140 W	FSM
Toaster	Fagor	220 V	650 W	ON/OFF
Coffee maker	Goodway	220 V	500 W	ON/OFF
Fruit mixer	Moulinex	220 V	400 W	ON/OFF
Bulb#1	—	220 V	25 W	ON/OFF
Bulb#2	—	220 V	40 W	ON/OFF
